# Machine learning prediction of customer satisfaction in fitness centres

**DOI:** 10.1038/s41598-026-55627-1

**Published:** 2026-06-20

**Authors:** Manuel Alonso Dos Santos, Jerónimo García Fernández, Carlos Pérez Campos, Jonathan Cuevas Lizama

**Affiliations:** 1 Department of Marketing and Market Research, University of Granada, Granada, Spain; 2 Administration Department, Universidad Católica de la Santísima Concepción, Concepción, Chile; 3 Physical Education and Sport, University of Seville, Seville, Spain; 4 Department of Physical Education and Sport, Valencia Catholic University Saint Vincent Martyr, Valencia, Spain

**Keywords:** Machine learning, Sports management, Satisfaction, Fitness centres, Artificial intelligence, Engineering, Mathematics and computing

## Abstract

Customer satisfaction in fitness centres is critical for fostering loyalty, higher spending, cross-buying, and positive recommendations. This study seeks to develop a predictive model of gym users’ satisfaction, identify its main determinants, and optimise predictive accuracy through machine learning techniques. Data from 10,368 users across five Spanish fitness centre chains were analysed. Five machine learning algorithms were applied: decision tree, random forest, logistic regression, gradient boosting, and Naïve Bayes. Model performance was evaluated using AUC, sensitivity, specificity, F-measure, Cohen’s Kappa, and overall accuracy. The random forest model showed the highest accuracy (AUC = 0.954, sensitivity = 0.933, specificity = 0.825, F-measure = 0.91, Cohen’s Kappa = 0.767, overall accuracy = 0.889). The most influential factors for satisfaction were the overall environment of the centre, employee trustworthiness, staff quality, and management of waiting times. This study extends prior research by applying machine learning algorithms to explain customer satisfaction in fitness centres, positioning satisfaction as the primary predictive outcome and providing interpretable insights into how environmental and service-related factors shape satisfaction beyond traditional retention-focused approaches.

## Introduction

The fitness centre sector has experienced significant growth globally, in the US and in Spain in recent years. Globally, the sports fitness centre market is expected to grow at a compound annual growth rate of 4.43% between 2023 and 2033, reaching a value of USD 372.86 billion by 2033^1^. In addition, global demand for fitness increased in 2023, with the US reaching a record penetration rate of 23.7% of the population enrolled in fitness centres^2^. In Spain, the gym and fitness centre sector had a turnover of €2,2 billion in 2022. The sector is expected to close 2025 with a turnover of over €1.5 billion, 15.4% more than in 2024^3^. The growth of the sector attracts more customers and implies more competition, but also leads to a higher churn rate and customer flight to competitors^4^.

Customer satisfaction is a key concept in the management of any service and, in the context of gyms and fitness centres, is critical to ensure member retention. Satisfaction is defined as the extent to which the performance of a product or service meets or exceeds customer expectations^5^ and as a pleasurable response to a good, service, benefit, or incentive^6^. In this sense, customer satisfaction in a gym relates to the user’s overall experience with the service received^7^, which implies that its evaluation is subjective and depends on the perception of quality, expectations, and previous experiences^8^.

From a strategic point of view, understanding which factors influence customer satisfaction not only improves service quality, but also increases the likelihood of retention^9^. In fact, research has shown that there is a relationship between perceived quality, perceived value, satisfaction, and customers’ future behavioural intention^10^. In other words, a satisfied customer is more likely to continue their membership and less likely to drop out.

One of the most widely used models to understand customer satisfaction is the disconfirmation paradigm, which is widely adopted in the marketing and fitness literature^11^. According to this approach, customers form expectations about employee behaviour and service quality, and their satisfaction is affected when these expectations are exceeded or not met^12^.

The problem of customer retention is a major concern for fitness centres, as several studies have identified multiple reasons why users abandon their membership^13^. Factors such as age and gender significantly influence the dropout rate^14^. Moreover, the lack of a clear retention strategy is a weakness in many companies in the sector^15^. It is therefore crucial to design specific strategies to avoid churn and maximise customer loyalty.

Previous research has identified several key elements to improve retention. For example, factors such as physical environment, communication, and accessibility play a determining role in membership renewal^13^. In this context, it is essential to identify less satisfied customers, understand their reasons for dissatisfaction and develop personalised strategies to improve their experience.

While prior machine learning studies in the fitness context have mainly focused on behavioural outcomes such as churn or membership duration, satisfaction reflects a more immediate psychological evaluation of the service experience. In this sense, satisfaction can be understood as a more proximal outcome that precedes behavioural responses such as renewal or dropout^8^. Focusing on satisfaction therefore adds conceptual value, as it allows earlier detection of dissatisfaction before it translates into observable customer behaviour.

Machine-learning machines can help identify dissatisfied customers with better accuracy than traditional techniques because they can handle unstructured data (text, audio, images), have the ability to learn and automatically adjust to new patterns without the need for manual reprogramming^16^, automatically extract relevant features, allow processing large volumes of data, are superior at capturing non-linear relationships^17^, and have greater resistance to overfitting through techniques such as regularisation and cross-validation^18^.

The uptake of artificial intelligence in the fitness industry is still very low despite its potential^19^. Machine-learning machines could predict seasonal behaviour and specific demand for services, automate marketing campaigns and churn prevention, provide 24/7 customer support, optimise pricing, or optimise resources among other examples^20^.

The aim of this research is to apply and evaluate different machine-learning (ML) algorithms to explain satisfaction of consumers in a fitness centre. Specifically, we seek to develop a predictive model, identify determinants, and optimise the accuracy of the model. This study endeavoured to answer the following research questions.

RQ1. Which ML model demonstrated the best performance in classifying user satisfaction of a fitness centre?

RQ2. What is the best predictor of satisfaction with the fitness centre?

The contributions of this research can be grouped into three categories: (1) Even with the importance of the sector, little research exists on user satisfaction with a fitness centre, and no other article is based on ML. (2) Most of the research on satisfaction has been approached from a linear methodology^21–23^, particularly structural equation modelling. The ML approach allows working with large volumes of data, captures non-linear and complex relationships, allows optimisation of the model, and does not have the limitation that linear models have for working with multidimensional data. (3) Finally, the ML methodology allows researchers to segment users. This advantage offers companies personalised strategies to improve satisfaction and retention in different demographic groups. The implementation described below makes it easy to bring it in-house.

## Literature review

### Perspectives on customer satisfaction of fitness centres

In the fitness sector, satisfaction has been agreed as the degree to which a product or service meets or exceeds customer expectations, as well as a pleasurable response to a good, service, benefit, or incentive. This evaluation is subjective and linked to the user’s overall experience with the service provided^5,24,25^.

Some work suggests that satisfaction is an affective response that stems from a cognitive judgement^26^. This perspective has gained support even among researchers who defend the disconfirmation paradigm as an explanatory core of satisfaction^27–29^.

Among the antecedents examined in the literature related to fitness centres, the influence of the servicescape stands out^30^. The servicescape refers to the physical environment where a service is provided and consumed, combining tangible elements that facilitate the interaction between the customer and the provider^31^. The servicescape is composed of environmental conditions such as lighting, temperature, noise, music, and aromas; the spatial layout and functionality composed of the arrangement of furniture, equipment, and spaces that facilitate or hinder customer objectives; and signage, symbols, and artefacts^32^. A reasonably similar approach was made by Sevilmiş et al.^13^ through physical environmental quality (visible physical facilities).

From an affective point of view, satisfaction has been addressed through E-life styles^9^, sense of community^24^, and member identity^33^.

Perhaps the most popular antecedent in the literature has been perceived quality^10^. Satisfaction has been closely related to service quality, although there is debate about the direction of this relationship^34^. Some authors consider satisfaction as an antecedent of perceived service quality^35^, while others argue the opposite^36^. However, the most widely accepted position today is that service quality is an antecedent of satisfaction^5,37^. Recent research has concluded that both service quality and customer satisfaction act as mediators of behavioural intentions^22^.

Trust has been defined as the level of reliability and integrity of facilities^7^. Consumer trust is based on the belief that the provider will act in their long-term interest and is directly linked to satisfaction^38^. Trust and customer satisfaction are related, as trust requires assessing the reliability and commitment of the other party^38^. In service marketing, trust is key to maintaining long-lasting relationships^39^. Building trust has been linked to the social and communication/listening skills of fitness instructors^11^, determining that customer/staff interaction leads to behaviour^40^. Not surprisingly, it is one of the three most cited determinants leading to satisfaction in the fitness-centre literature^5^.

Finally, the relationship between perceived value and satisfaction has been verified in the literature^10^. Studies in the fitness industry that examined their relationship are scarce^39^, but a positive and significant relationship has been found in diverse contexts and cultures^41–43^. Although the construct of perceived value was addressed in a unidimensional way with a focus on value for money^37,42^, some scholars believe that its multi-dimensional (economic, emotional, and social) structure should be considered^39^. In this respect, scholars have not yet reached a consensus, so debates in this area are still ongoing.

Regarding the consequences of satisfaction, customer satisfaction plays a crucial role in the success of low-cost fitness clubs^24^. Several authors have examined the influence of satisfaction on customer retention^5^, intention to renew^23^, intention to attend^33^, intention to recommend^22^, or intention to engage in physical activity^25^. But only a couple of authors have applied artificial intelligence to the fitness centre sector^19^ and specifically to consumer behaviour^17^. No scientific article has examined the determinants of satisfaction.

### KNIME analytics platform

KNIME Analytics Platform is an open-source data analytics tool that allows users to design and execute Machine Learning (ML) workflows without programming^44^. Its modular architecture allows for flexible and scalable analytical processes that can be integrated with languages and tools such as Python, R, and Spark, making it ideal for data scientists and analysts in several areas^45,46^. Its visual approach facilitates data manipulation, model selection, hyperparameter fitting, and interpretation of results, making it an accessible solution for both beginners and experts in data science. In marketing, KNIME is applied to several key areas, such as churn prediction, sentiment analysis, search engine optimisation (SEO), and customer experience evaluation^47^. The platform allows integrating and analysing data from multiple sources, building ML models and automating processes, and improving data-driven decision making.

KNIME is based on the use of nodes, which are the fundamental elements for processing data. Each node performs a specific function, such as selecting rows, performing aggregations, or training machine learning models. These nodes are incorporated into the Workflow Editor from the Node Repository and can have three different states: red if they have not been configured, yellow if they are configured but have not yet been executed, and green if they have completed their execution successfully. To function, each node requires a previous configuration with specific parameters and must be connected to other nodes within the workflow.

The pipeline in KNIME is built by linking nodes in a logical sequence. Typically, it starts with a data import node, such as a file reader or a database connector. The data then goes through cleansing and normalisation processes before being split into training and test sets by a partitioning node.

The connection between nodes is established via ports, which vary in shape and colour depending on the type of data they handle. Generally, input ports are placed on the left side of the node and output ports on the right. Triangles represent tabular data, squares may contain models, images or connections, while circles usually transmit variables.

In addition, KNIME allows nodes to be grouped into meta-nodes and components, making it easy to organise and reuse entire sections of the workflow. This is especially useful for recurring processes, such as the calculation of key performance indicators (KPIs).

## Method

This study proposes an AutoML-based approach to classify fitness centre users according to their level of satisfaction and to analyse the features that influence this classification. AutoML systems have revolutionised machine learning by automating key tasks such as model selection, hyperparameter tuning, and the identification of relevant features, speeding up their application in different domains. Within this process, feature selection plays an essential role, as it contributes to improving model accuracy, reducing the risk of overfitting, and facilitating the handling of high-dimensional data. Its main focus is to select variables with a strong relationship to the target variable, thus avoiding redundancies. Recent research has demonstrated its impact in areas such as data mining and intrusion detection, highlighting its usefulness in model optimization^48^.

The process starts with data collection and pre-processing, then breaks down into training and test sets to ensure proper validation of the models. The AutoML node in KNIME automates the process of building, optimising, and evaluating machine learning models, allowing the best model to be selected without the need for manual adjustments. It works in several stages. First, the data preprocessing stage involves data cleaning normalisation and the preparation of the dataset prior to model training. Then, it tests different machine learning algorithms to determine which one offers the best performance. It then automatically adjusts the hyperparameters of each model to improve its accuracy. Once the optimisation is complete, the node evaluates the performance of the models using metrics such as accuracy, precision, recall, and F1-score, selecting the most suitable one^46^.

Data were randomly divided into training and testing sets using an 80 20 split. Model optimisation was conducted through the KNIME AutoML framework which integrates automated hyperparameter tuning and internal resampling procedures during model selection. This approach allows multiple algorithmic configurations to be evaluated while minimising overfitting risk and enhancing the robustness and generalisability of predictive performance. Model performance was evaluated on a held-out test set to ensure an unbiased estimation of predictive accuracy.

**Fig. 1:**
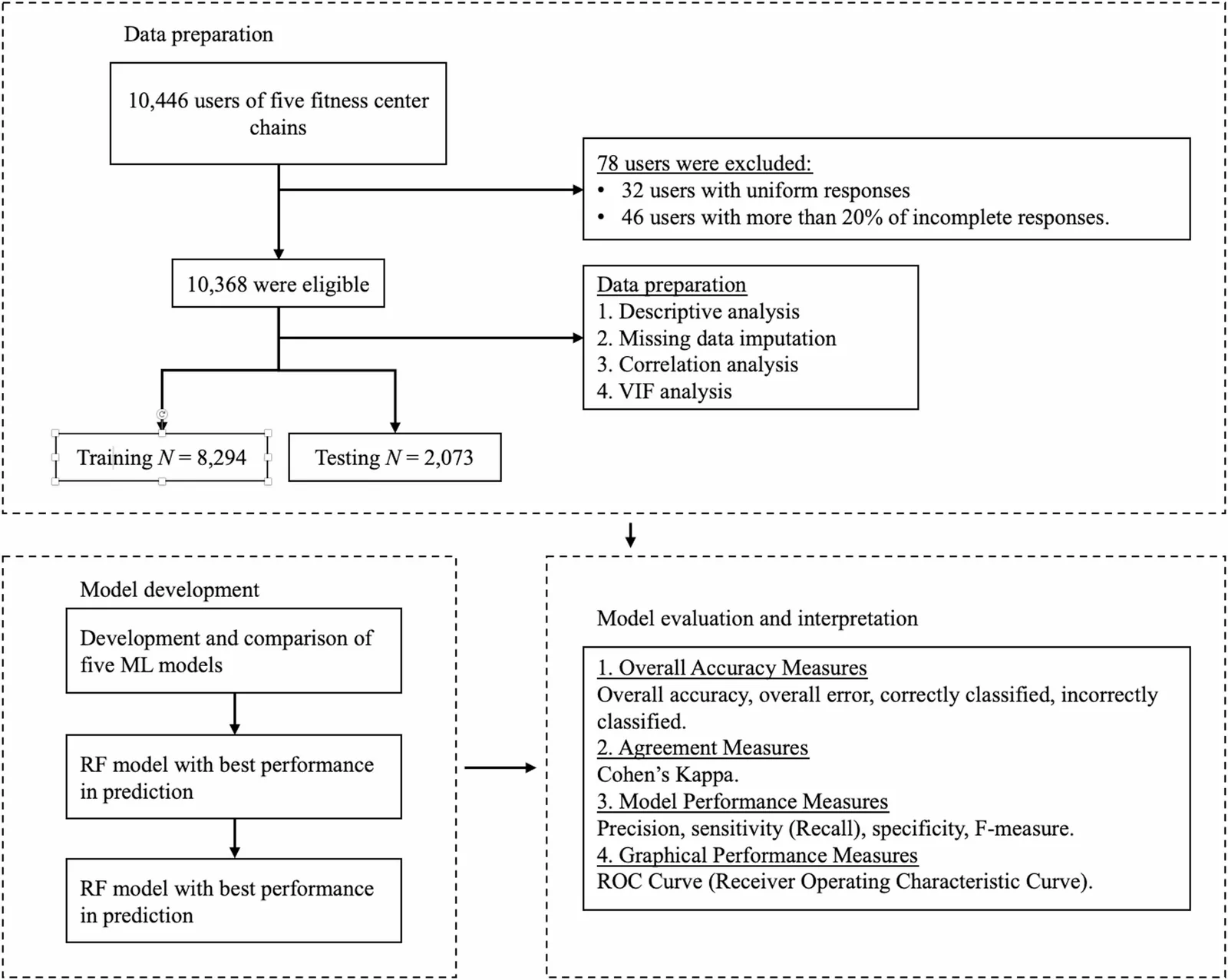
Adapted from: Bian and Cork^51^.

In addition, AutoML in KNIME includes a feature analysis, identifying the most influential variables in the predictions. Its internal structure is composed of multiple sub-processes working together, including modules for model selection, hyperparameter optimisation, and performance evaluation, which facilitates workflow automation in machine learning tasks^48–50^. The whole pipeline in KNIME is simplified in Fig. 1.

Model optimisation was performed within the AutoML node in KNIME, where hyperparameters were automatically selected. The resulting Random Forest configuration included 100 trees and a minimum node size of 1, while tree depth was not explicitly constrained, allowing the algorithm to determine the optimal structure during training. Global feature importance was derived from the AutoML surrogate models for interpretability purposes. Independently from the AutoML optimisation, the predictive performance of the Random Forest model was validated using stratified k-fold cross-validation (k = 10) implemented through the X-Partitioner and X-Aggregator nodes in KNIME. This procedure was applied as an external validation step and did not inform model selection, which was conducted exclusively within the AutoML framework, thereby avoiding optimistic bias in performance estimation.

### Data collection

The surveys were distributed among 56 fitness centres located in Spain, belonging to five different chains. After obtaining management consent, an online questionnaire was administered using Google Forms over five months, ensuring voluntary participation and confidentiality. The estimated completion time was 10 min.

The study protocol was reviewed by the Ethics Committee on Human Research (CEIH) of the University of Granada, which confirmed that it was exempt from formal ethical review (waiver of ethical approval). The study involved the participation of adults through anonymous surveys, and all participants provided their informed consent on a voluntary basis. According to the institutional and ethical regulations of the University of Granada (Spain), this type of observational and non-interventional study does not require formal ethical approval, as it did not involve clinical procedures, sensitive personal data, or vulnerable populations. Data were collected anonymously and treated confidentially, in accordance with basic ethical principles of scientific integrity and respect for participants. The research was conducted in accordance with the ethical principles of the Declaration of Helsinki and the European General Data Protection Regulation.

All included variables were assessed using multi-item scales validated in previous studies, using a Likert scale from 1 to 7. In particular, the measurement of the servicescape variable was based on 21 items developed by Kim et al.^30^. The perceived quality scale was adapted from Brady and Cronin^52^, later implemented in Nuviala et al.^53^ or Reyes Robles et al.^54^. The loyalty scale was adapted from Zeithaml et al.^55^. The employee quality scale was adapted from Lam et al.^56^. The perceived quality scale was adapted from Rial et al.^57^ (hereafter IPAV). The perceived value scale was adapted from Zeithaml^58^.

To adapt the questionnaire to the Spanish context, the questionnaire was translated and synthesised following Martín-Consuegra et al.^59^, ensuring accuracy and semantic equivalence. These variables were collected based on Clavel San Emeterio et al.^60^.

The dependent variable was Satisfaction (“I am satisfied with the sports services of this fitness centre”), measured on a 1–7 scale. It was recoded into a binary outcome because the study was designed as a classification problem aimed at distinguishing satisfied from non-satisfied users for managerial decision-making. Users scoring above the median (> 5.5) were classified as satisfied, whereas users scoring below this threshold were classified as not satisfied; this procedure is consistent with previous classification-based approaches using satisfaction-related outcomes^61–63^. We acknowledge that dichotomisation entails a loss of information. Therefore, alternative thresholds (≥ 5 and ≥ 6) were tested as robustness checks.

Table 1 presents a brief statistical description of the demographic and fitness centre usage variables. Appendix 1 presents the variables in the database and their descriptions.

**Table 1:** Descriptive statistics.

Variable	Codes	Frequency	Percentage
Gender	Male	5,864	56.5
	Female	4,504	43.4
Age	20 and less	388	3.7
	21–30	2,781	26.8
	31–40	2,970	28.6
	41–50	2,750	26.5
	51–60	1,168	11.2
	Above 60	311	3
Membership	3 months and less	1,469	14.1
	3–6 months	1,510	14.5
	6–12 months	1,810	17.4
	1–2 years	2,668	25.7
	Above 2 years	2,911	28.0
Attendance	Less than 1 time per week	249	2.4
	1 time	385	3.7
	2 times	2,015	19.4
	3 times	4,236	40.8
	4 times	3,483	33.5
	Above 4 times	249	2.4
Fitness chain	1	3,755	36.2
	2	1,095	10.5
	3	1,708	16.4
	4	710	6.8
	5	3,100	29.8

### Preprocessing procedures and feature selection

KNIME begins by importing data through nodes such as the CSV Reader or Excel Reader. Next, preprocessing steps such as cleaning, normalisation, and transformation are applied because their use significantly improves the prediction outcome^64^. The inclusion of many predictors in a model to solve a classification problem presents serious theoretical disadvantages such as: (i) overfitting, (ii) correlation issues, (iii) difficulty in interpreting results, and (iv) a slower training process. The idea is to reduce noise and redundancy in the final model. In fact, the principle of parsimony states that the best statistical model has fewer parameters (variables) and lower dimensionality^65^.

First, we performed an exploratory data analysis (EDA) to analyse and investigate the datasets and summarise their main characteristics using data visualisation methods. This helped determine the best way to manipulate the data sources, facilitating the discovery of patterns and the detection of anomalies.

Second, the correlation between features was calculated using the “Linear correlation” node along with the “Correlation filter” node for automatic feature selection in the case of highly correlated variables^66^. A construct should be removed from the dataset if two constructs are highly correlated^67^. We have set a maximum correlation filter with a coefficient of 0.8 to identify and remove highly correlated variables. As a result, 11 predictors were excluded from the dataset following the correlation filter procedure see Appendix 1 where the removed variables are indicated.

Following the correlation-based filtering, several excluded variables corresponded to constructs conceptually proximate to the dependent variable, such as loyalty and recommendation measures, which capture post-consumption evaluations closely related to satisfaction. Their exclusion is therefore not only statistically driven, but also conceptually consistent, as retaining such variables could introduce redundancy and blur the distinction between predictors and outcome.

Third, unlike what is often claimed, correlation does not necessarily mean multicollinearity as they are not the same. Thus, multicollinearity problems cannot be analysed by using the correlation matrix but by using the Variation Inflation Factor VIF KNIME VIF node^68^. The variable SC7 presented the highest VIF value 4.13 confirming the absence of multicollinearity problems VIF < 5 normally regarded as a critical cut-off value^69^. Therefore, no variables were removed based on the VIF analysis and all predictors were retained for subsequent model training.

Fourth, the dataset was randomly divided into training and testing sets using the partition node, with 80% used for training (8,294 users) and 20% used for testing (2,073 users). After this split, the predictor variables were standardised in the range [0,1]. The scaling parameters were estimated exclusively from the training set and subsequently applied to the test set, thereby preventing data leakage. The dependent variable, Satisfaction, was not standardised, as it was used as the binary classification target.

### Classification models

For classification purposes, decision tree (DT), random forest (RF), logistic regression (LR), gradient booster (GD), and Gaussian Naïve Bayes (NB) classifiers were applied. A brief introduction is given below:

The selected algorithms (decision tree, random forest, logistic regression, gradient boosting, and Gaussian Naïve Bayes) were chosen for their ability to handle nonlinear relationships, prioritise key variables, and provide interpretability. Specifically, the decision tree enables the generation of interpretable rules, as demonstrated by Tsami et al.^70^ in public transportation, identifying critical variables such as exterior design and cleanliness with 89.5% accuracy.

Logistic regression quantifies impacts through odds ratios, validated in recent studies analysing consumer behaviour in services and payment methods^72,73^, achieving reliability above 89%. Gradient boosting (XGBoost) effectively handles imbalanced data^51^. Finally, Gaussian Naïve Bayes provides computational efficiency, reaching 87% accuracy in airline satisfaction prediction^74^.

This combination integrates different approaches: logistic regression for marginal effects, random forests for predictive accuracy, and decision trees for actionable rules—a methodology validated in sectors such as healthcare, transportation, and retail.

## Results and discussion

### Results of correlation and PCA method

**Fig. 2:**
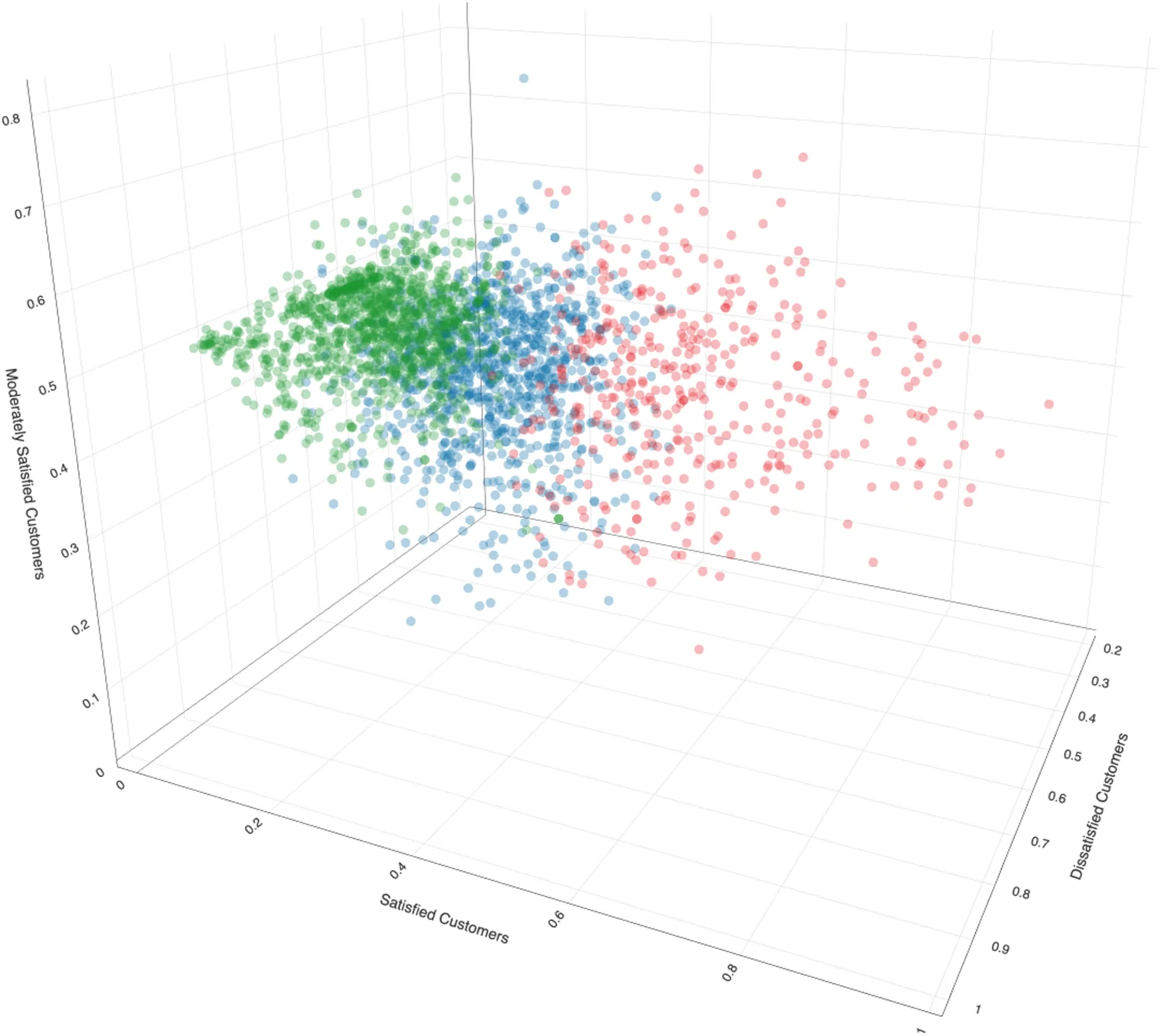
Three-dimensional scatterplot of customer satisfaction by cluster. Cluster 0 is shown in green, Cluster 1 in red, and Cluster 2 in blue.

A K-means clustering analysis was performed, restricting the number of clusters to a small range of plausible solutions (k = 2–3) and selecting the final configuration (k = 3) based on the interpretability and stability of the resulting segmentation. A deterministic initialisation was used to ensure reproducibility. The analysis identified three distinct clusters, with significant differences in sociodemographic variables and service perception. These groups, labelled as “Satisfied Customers” (Cluster 0), “Moderately Satisfied Customers” (Cluster 1), and “Dissatisfied Customers” (Cluster 2). Figure 2 shows a 3D PCA with three clusters (green, blue and red) representing different levels of customer satisfaction. The dots are distributed among the three categories, indicating clear clustering patterns.

To evaluate the differences between clusters, one-way analyses of variance (ANOVA) were performed. The results showed statistically significant differences between the three clusters on all variables analysed (*p* < .001). Tukey’s post-hoc tests revealed that the clusters differed from each other on all variables (*p* < .001), while clusters 1 and 2 showed significant differences on most, but not all variables (*p* < .05).

The effect size, calculated using partial eta squared (η²), indicated large effects for overall satisfaction (η² = 0.68), loyalty (η² = 0.72), and perceived service quality (η² = 0.65). Effects were moderate for perceived servicescape (η² = 0.48) and importance attributed to different aspects of the service (η² = 0.39).

Users in Cluster 0 (named Satisfied Customers − 45%) have an average age of 35 years (relatively younger), an attendance frequency of 4 times per week (relatively higher), a permanence of 3 years (relatively longer), and an experience of 4 years (relatively higher). This group is characterized by high ratings in several key areas. Users highlight the friendliness and treatment by the staff (0.38, IPAV1), as well as the professionalism (0.44, IPAV2) and effectiveness of the instructors (0.53, IPAV3). Additionally, they positively value the personalized service (0.76, IPAV4) and the interest shown by the staff for customer continuity (0.29, IPAV5). Regarding the environment, users appreciate the physical environment, lighting, and decoration (0.88, IPAV6), as well as the sports spaces (0.74, IPAV7) and cleanliness and hygiene (0.90, IPAV8). They also consider the sports equipment (0.90, IPAV9) and the changing rooms, toilets, and showers (0.91, IPAV10) to be in good condition. Overall, users in this group have a very positive perception of the signs and posters in the fitness centre (0.86, SC1), the distribution of the fitness centre (0.92, SC2), and the exterior design (0.90, SC5). Additionally, they value the quality of the equipment (0.90, SC8) and the complementary facilities (0.66, SC10). The background noise level (0.90, SC13) and the fitness centre environment (0.95, SC16) are also well-rated, as well as the music used (0.90, SC15) and the lighting levels (0.94, SC17). Finally, they consider the temperature and humidity (0.88, SC18) to be pleasant.

Users in Cluster 1 (named Moderately Satisfied Customers − 35%) have an average age of 40 years (average age), an attendance frequency of 3 times per week (average frequency), a permanence of 2 years (average permanence), and an experience of 3 years (average experience). This group presents moderate ratings in the same key areas as Cluster 0. Users rate the friendliness and treatment by the staff (0.51, IPAV1), the professionalism (0.47, IPAV2) and effectiveness of the instructors (0.68, IPAV3), and the personalized service (0.74, IPAV4) as acceptable. They also consider the interest shown by the staff for customer continuity (0.21, IPAV5) to be adequate. In terms of the environment, users moderately rate the physical environment, lighting, and decoration (0.43, IPAV6), the sports spaces (0.29, IPAV7), and the cleanliness and hygiene (0.45, IPAV8). The sports equipment (0.51, IPAV9) and the changing rooms, toilets, and showers (0.41, IPAV10) also receive acceptable ratings. Users in this group have a moderate perception of the signs and posters in the fitness centre (0.74, SC1), the distribution of the fitness centre (0.57, SC2), and the exterior design (0.58, SC5). The quality of the equipment (0.63, SC8) and the complementary facilities (0.67, SC10) are also rated as acceptable. The background noise level (0.76, SC13) and the fitness centre environment (0.71, SC16) receive moderate ratings, as well as the music used (0.09, SC15) and the lighting levels (0.74, SC17). Finally, they consider the temperature and humidity (0.54, SC18) to be adequate.

Users in Cluster 2 (named Dissatisfied Customers − 20%) have an average age of 45 years (relatively older), an attendance frequency of 2 times per week (relatively lower), a permanence of 1 year (relatively shorter), and an experience of 2 years (relatively lower). This group is characterized by lower ratings compared to the other groups. Users in this group have a less favourable perception of the friendliness and treatment by the staff (0.45, IPAV1), the professionalism (0.44, IPAV2) and effectiveness of the instructors (0.60, IPAV3), and the personalized service (0.74, IPAV4). They also consider the interest shown by the staff for customer continuity (0.25, IPAV5) to be lower. Regarding the environment, users rate the physical environment, lighting, and decoration (0.70, IPAV6), the sports spaces (0.46, IPAV7), and the cleanliness and hygiene (0.71, IPAV8) less favourably. The sports equipment (0.70, IPAV9) and the changing rooms, toilets, and showers (0.74, IPAV10) also receive lower ratings. Users in this group have a less favourable perception of the signs and posters in the fitness centre (0.66, SC1), the distribution of the fitness centre (0.75, SC2), and the exterior design (0.72, SC5). The quality of the equipment (0.36, SC8) and the complementary facilities (0.70, SC10) are also rated less favourably. The background noise level (0.79, SC13) and the fitness centre environment (0.69, SC16) receive lower ratings, as well as the music used (0.79, SC15) and the lighting levels (0.70, SC17). Finally, they consider the temperature and humidity (0.54, SC18) to be less pleasant.

### Performance of classification models

The model evaluation was carried out using a confusion matrix, a tool that analyses the performance of classification models. This matrix breaks down the total predictions into four categories: users who are not satisfied (labelled as 0) and correctly classified as such are referred to as true negatives (TN). If these users were incorrectly classified as satisfied, they are considered false positives (FP). Similarly, users who are satisfied (labelled as 1) and correctly identified are recorded as true positives (TP), while cases where these users were mistakenly classified as not satisfied are false negatives (FN).

To assess and compare the performance of machine learning models, various metrics were used (see Table 2), such as the area under the curve (AUC), ROC curve, Cohen’s Kappa coefficient, precision, sensitivity, specificity, and F-measure (Table 2). An ideal model typically has an AUC value close to 1, indicating excellent class differentiation ability. These metrics depend directly on the correct classification of true positives, false positives, true negatives, and false negatives. The ROC curve, in particular, provides a visualisation of the trade-off between the true positive rate and the false positive rate, with its area serving as a reliable indicator of model quality.

When comparing decision trees (DT), random forests (RF), logistic regression (LR), Naïve Bayes (NB), and Gradient Boosting (GB) models, it was observed that the random forest (RF) model achieved the best performance across several key metrics. The highest overall accuracy was achieved by RF (0.889), followed by GB (0.887), DT (0.882), LR (0.877), and finally, NB, which had the lowest performance (0.836). This pattern was also reflected in the error rate, where RF had the lowest value (0.110), compared to GB (0.113), DT (0.118), LR (0.123), and NB (0.164).

Cohen’s Kappa coefficient, which measures the agreement between predictions and actual values beyond chance, showed that RF had the highest agreement with a value of 0.767, followed by GB (0.764), DT (0.752), and LR (0.741). In contrast, NB showed the lowest agreement, with a value of 0.654. These values reinforce the reliability of RF and GB as robust and consistent classification models.

The area under the ROC curve, which balances sensitivity and specificity, showed that RF and GB excelled with a value of 0.954, surpassing LR (0.943), DT (0.925), and NB (0.912). This confirms the superior ability of RF and GB to distinguish between classes.

In terms of precision, which measures the proportion of correct positive predictions, the GB model performed best (0.894), followed by RF (0.889), DT (0.888), LR (0.878), and NB (0.846).

Sensitivity, which is crucial in applications where identifying all positive instances is essential, was highest for RF (0.933), followed by DT (0.918), LR (0.923), and GB (0.921), while NB had the lowest value (0.886). Regarding specificity, which evaluates the model’s ability to correctly identify negative cases, GB led with 0.836, followed by DT (0.827), RF (0.825), LR (0.809), and NB (0.761).

Finally, the F-measure, which combines precision and sensitivity, showed that RF had the best performance (0.911), followed by GB (0.907), DT (0.903), LR (0.901), and NB (0.866).

In conclusion, the random forest (RF) model stands out as the best overall choice, demonstrating excellent classification ability across multiple metrics, closely followed by the Gradient Boosting (GB) model. Both models represent solid options for machine learning applications in binary classification of satisfied and non-satisfied users.

**Table 2:** Confusion matrix´s indicators.

	Decision Tree	Random Forest	Logistic Regression	Naive Bayes	Gradient Boosting
Overall accuracy	0.882	**0.889**	0.877	0.836	0.887
Overall error	0.118	**0.11**	0.123	0.164	0.113
Cohen´s Kappa	0.752	**0.767**	0.741	0.654	0.764
Correctly classified	1847	**1863**	1837	1750	1858
Incorrectly classified	247	**231**	257	344	236
ROC_Curve	0.925	**0.954**	0.943	0.912	**0.954**
Precision	0.888	0.889	0.878	0.846	**0.894**
Sensitivity	0.918	**0.933**	0.923	0.886	0.921
Specificity	0.827	0.825	0.809	0.761	**0.836**
F-measure	0.903	**0.911**	0.901	0.866	0.907

Bold values indicate the best-performing model for each evaluation metric.

To further assess model robustness beyond a single data split, a k-fold cross-validation procedure was conducted using the X-Partitioner and X-Aggregator nodes in KNIME. The cross-validated Random Forest achieved an aggregated accuracy of 83.16% (Cohen’s κ = 0.648), indicating substantial agreement and stable predictive performance across validation folds. As expected, this cross-validated accuracy is more conservative than the optimised model performance, reflecting a stricter evaluation across multiple training and validation partitions rather than a single holdout configuration.

### Sensitivity to dichotomisation threshold

As a robustness check, we evaluated the sensitivity of the results to alternative dichotomisation thresholds of the satisfaction variable. When applying a more lenient threshold (≥ 5), model accuracy increased to 0.906, driven by a rise in sensitivity (0.957), but at the cost of a reduction in specificity (0.752), as a larger proportion of cases were classified as satisfied. Conversely, using a stricter threshold (≥ 6) led to higher specificity (0.879) but a decrease in sensitivity (0.887), due to the reduced number of satisfied cases. Importantly, despite these variations in absolute performance metrics, the relative ranking of the models remained unchanged, with Random Forest consistently outperforming alternative classifiers. Likewise, the identification of the most important predictors remained stable across all specifications, supporting the robustness of the main results.

### Global feature importance, surrogate random forest

Feature importance was automatically generated within the KNIME AutoML workflow and derived from tree-based surrogate models including Random Forest and Decision Tree using internal split-based importance measures rather than permutation or SHAP based approaches.

A global surrogate RF approximates the predictions of the original model. It is trained on pre-processed input data with optimized parameters: “Tree Depth”, “Number of models”, and “Minimum child node size”. The surrogate model achieved 0.982 accuracy in predicting the target class “Satisfaction: 1”. Feature importance is determined by how often a feature is used for splitting and its rank within the trees. Higher values indicate greater importance.

Based on the RF global importance values, the most important variables are SC16 (the overall environment of the sports centre) that stands out with the highest importance score of 2.51, indicating its significant impact. Following closely is QE6 (the behaviour of the employees generates trust in the customer) with an importance score of 2.13, highlighting the crucial role of employee quality. SC8 (the quality of the equipment used) also shows high importance with a score of 1.68, emphasizing the relevance of this variable. SC4 (the sports centre is designed to minimize waiting time) has an importance score of 1.57, further underscoring its importance. SC11 (the equipment and facilities are safe) and SC6 (the interior design of the sports centre is pleasant) have importance scores of 1.32 and 1.16, respectively, indicating their substantial contributions. The least important variables are Fitness experience (importance: 0.00), Seniority of member (importance: 0.00), and chain (importance: 0.00). These importance values should be interpreted as relative contributions within the model rather than as absolute effect sizes. In particular, split-based importance measures are influenced by the correlation structure among predictors and may distribute importance across related variables. Therefore, the results reflect patterns associated with predictive performance rather than causal relationships.

### Global feature importance, surrogate decision tree

**Fig. 3:**
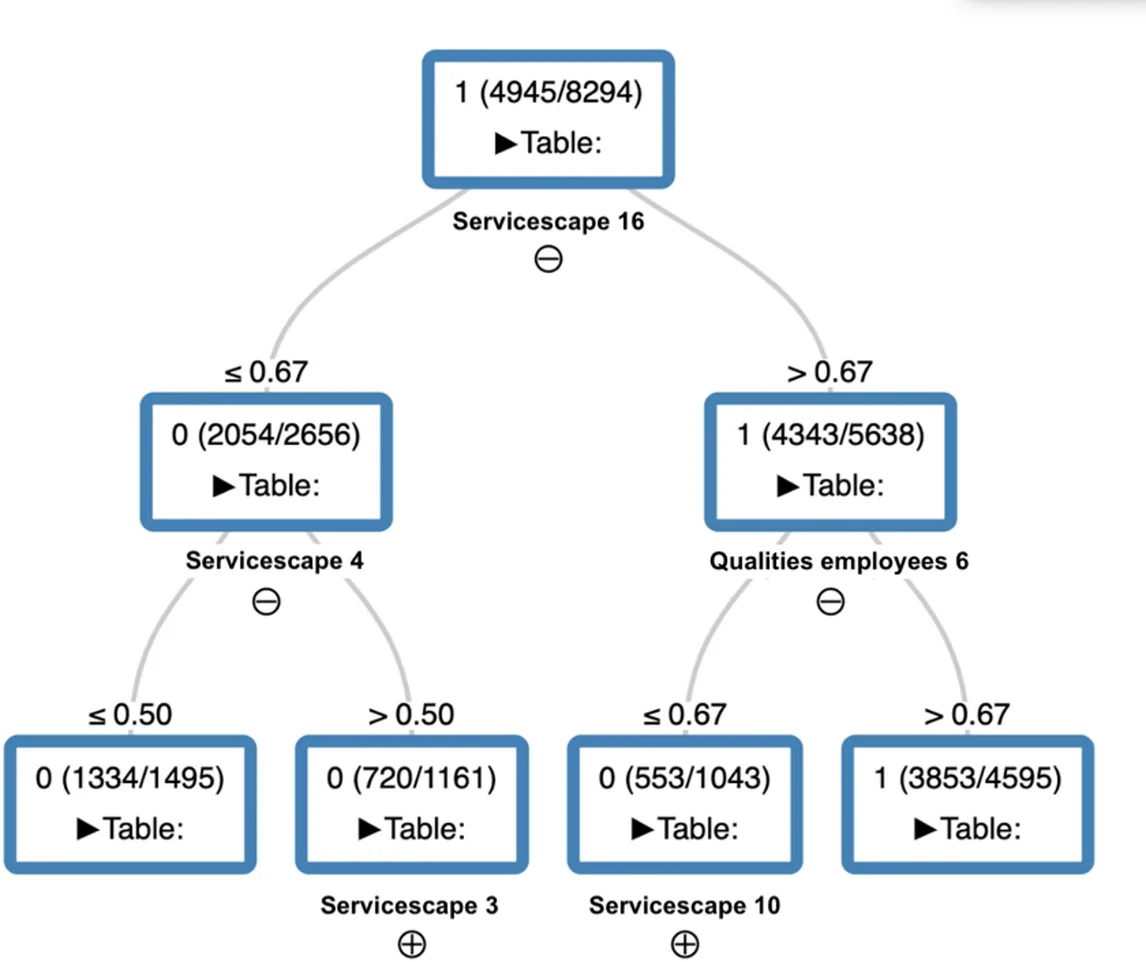
Surrogate decision tree.

Similarly to the previous model, the global surrogate DT approximates the predictions of the original model and provides an interpretable representation of the learned decision logic. It is trained on pre-processed input data with an optimized “Min number of records per node = 500” parameter. The surrogate model achieved 0.929 accuracy in predicting the target class “Satisfaction: 1”. Figure 3 visualizes the tree’s decision process, where top features have higher importance than those at the bottom. In particular, the first split is driven by SC16, highlighting the dominant role of the physical environment in determining customer satisfaction. Subsequent branches incorporate variables such as SC4, QE6, and SC10, which refine the classification boundaries through threshold values (e.g., 0.67 and 0.50). Unlike global importance rankings, this hierarchical representation illustrates how environmental perception acts as the primary decision driver, while employee related attributes and operational aspects contribute to secondary refinements in the prediction process, thereby providing additional interpretability beyond the quantitative metrics reported in the model results.

## Conclusions and recommendations

The results of this study provide valuable insights into the determinants of user satisfaction in fitness centres, using advanced machine learning techniques. The k-means analysis revealed three distinct groups of customers: Satisfied (45%), Moderately Satisfied (35%), and Dissatisfied (20%). This segmentation provides a basis for personalised marketing and service strategies, similar to what was proposed by Paschalidou et al.^75^ in their study on fitness centre customer segmentation.

The results are in line with previous research that identified dissatisfied occasional customers as dropouts at a significantly higher percentage than the sample as a whole^4^. Low engagement and high turnover mean that the group of ‘occasional dissatisfied customers’ should be a priority for fitness centre managers. Satisfaction is a significant antecedent of retention^76^. High dropout rates exemplify the importance of satisfaction and attendance behaviours in fostering member retention. Therefore, the results support previous work emphasising satisfaction (e.g. 75) and attendance (e.g. 59,76).

With respect to prediction model fit, the Random Forest model proved to be the most accurate predictor of satisfaction, achieving an AUC of 0.954, a sensitivity of 0.933 and a specificity of 0.825. This exceeds the results obtained in previous studies, such as that of Li et al.^71^, which achieved an AUC of 0.932 in the context of hospitality satisfaction.

Compared with previous research in the fitness centre literature based on structural equation modelling including covariance-based SEM and PLS SEM approaches^10,22,24,37,40^ the present study highlights a shift from explanation-oriented modelling toward prediction-oriented approaches. Earlier SEM based studies typically reported moderate explanatory power with R squared values for satisfaction and behavioural intentions generally ranging between approximately 0.30 and 0.60 reflecting explained variance rather than predictive discrimination. In contrast the Random Forest model achieved an AUC of 0.954 and an overall accuracy of 0.889 indicating strong predictive capability in distinguishing satisfied from non-satisfied users. Although R squared values and predictive metrics such as AUC or accuracy are not directly comparable because they capture different modelling objectives this contrast illustrates how machine learning techniques can complement traditional SEM approaches by providing higher predictive precision while SEM models remain essential for theory testing and causal interpretation within fitness service research.

The most important variables predicting satisfaction were: The general atmosphere of the sports centre (SC16), the behaviour of the employees that generates customer trust (QE6), and the quality of the equipment used (SC8).

These findings are in line with previous research that has highlighted the importance of the physical environment and service quality in customer satisfaction. For example, Garcia-Fernandez et al.^37^ found that service convenience and perceived quality significantly influence satisfaction and loyalty in low-cost fitness centres. They are also consistent with recent evidence based on NLP and sentiment analysis, which identified the work environment and management-related dynamics as central factors in satisfaction formation^81^.

This manuscript confirms the successful interest in the literature in the influence of employee trust and equipment quality^5^. However, the general environment of the fitness centre has received limited attention in the literature^78^, despite its strong influence in our findings. Although previous studies have considered it as an antecedent of satisfaction, its relative importance has not been emphasised to the extent suggested by our results^79^.

### Implications for management

Some management implications for fitness centre managers that can be deduced from this study concern strategies and actions to help a fitness centre to be liked by users. Firstly, it is crucial to improve the overall ambience of the fitness centre, as it was identified as the most important predictor of customer satisfaction. Managers should invest in improving aspects such as lighting, music, and interior design to create a more pleasant atmosphere. This is in line with the findings of Ong and Yap^80^, who highlighted the importance of the servicescape in the emotional responses and behaviour of customers in fitness centres.

Secondly, it is recommended to focus on staff training, particularly on skills that build customer confidence. This could include training programmes in customer service and relationship building. This recommendation aligns with the findings of Garcia-Fernandez et al.^37^, who found that service quality significantly influences customer satisfaction and loyalty in low-cost fitness centres and specifically the improvement of staff^33^.

Third, it is important to maintain and regularly update the equipment of the centre, as the quality of the equipment was found to be a significant factor in customer satisfaction^43^. This is in line with the findings of Foroughi et al.^22^, who highlighted the importance of the quality of the main product on the emotions and behaviours of sports centre users. Following this idea, innovation in service development could also lead to better evaluation of equipment. For example, Wang and Chiu^40^ highlighted the importance of service innovation on membership renewal intention in fitness centres.

Fourth, it is suggested to optimise processes to minimise waiting times, which can be achieved through efficient facility design and customer flow management systems. This recommendation is based on the identified importance of the SC4 variable in the predictive model and aligns with the research of Garcia-Fernandez et al.^37^ on service convenience in fitness centres.

Finally, it is recommended to develop personalised strategies based on the customer segmentation identified in the study (Satisfied, Moderately Satisfied and Dissatisfied). This would address the specific needs of each group and improve their overall experience. This recommendation is supported by the segmentation approach proposed by Paschalidou et al.^75^ to improve loyalty and word-of-mouth communication in fitness centres.

## Limitations and future research directions

This study provides valuable insights into customer satisfaction in fitness centres using machine learning techniques. However, several limitations must be acknowledged, which also point to promising avenues for future research. The current database lacks variables related to pricing, a crucial factor in customer satisfaction and retention. The study focused on fitness centres in Spain, which may introduce cultural bias and potentially limit the generalisability of the findings to other cultural or international contexts. In addition, the analysis uses cross-sectional data, which provides only a snapshot of customer satisfaction. Satisfaction was also measured using a single-item indicator, which may not fully capture its multidimensional nature, and subsequently dichotomised for classification purposes, implying a loss of information that may affect both predictive performance and substantive interpretation. Future research should complement this classification approach with regression-based machine learning models, such as Random Forest Regressor or Gradient Boosting Regressor, to predict the original satisfaction score and compare whether the same predictors remain relevant when the full ordinal range of the dependent variable is retained.

Furthermore, although the dataset is large, it does not include actual behavioural outcomes (e.g., retention or spending), limiting the ability to directly link predicted satisfaction with observed customer behaviour.

Future research directions could focus on exploring more advanced artificial intelligence techniques, such as deep learning or natural language processing, to analyse unstructured data such as customer reviews or social media comments. Other research directions could include sentiment analysis, integrating data from wearable devices, connecting and tracking real-time usage of equipment installed in the centre, and implementing chatbots to measure real-time service satisfaction. By addressing these limitations and exploring these future directions, researchers can further improve our understanding of customer satisfaction in fitness centres and provide more actionable information for industry professionals.

## Data Availability

The datasets generated and/or analysed during the current study are available from the corresponding author upon request.

## Appendix 1

See Table 3.

**Table 3:** Definition of data set variables (FC: Fitness centre).

Variables	Description
Gym chain	Fitness centre chain (5 franchises)
Gender	The gender of the customer
Age	The age of the customer
Seniority of member	Registered at fitness centre
Frequency of attendance	Frequency attending the sports centre
Fitness experience	Experience in other competing fitness centres
Recommendation (NPS)	Likelihood of recommending current fitness centre
Perceived quality	
IPAV1	Friendliness and treatment of the staff
IPAV2	Professionalism of the instructors
**IPAV3**	**Effectiveness of the instructors**
IPAV4	Personalised service
IPAV5	Interest shown by staff in customer continuity
IPAV6	Physical environment, lighting, decoration
IPAV7	Sports facilities (fitness, supervised activities)
IPAV8	Hygiene and cleanliness
IPAV9	Sports equipment
IPAV10	Changing rooms, toilets and showers
Quality-Employees	
**QE1**	**Employees are always ready to help the customer.**
**QE2**	**Employees treat the client in a friendly and respectful way.**
QE3	Employees show the necessary knowledge to advise the client (exercise routines, use of equipment…)
**QE4**	**Employees show the specialised skills to perform their work.**
**QE5**	**Employees respond promptly to client requests**
QE6	The behaviour of the employees generates confidence in the client.
Servicescape-Signage	The signs and posters on the FC are useful.
Servicescape-Facility layout	
SC1	The FC is comprehensive.
SC2	The FC is designed for all skill levels.
SC3	The FC has more than enough space for me to feel comfortable.
SC4	The FC is designed to minimise my waiting time.
Servicescape-Facility design	
SC5	The exterior design of the FC is pleasing.
SC6	The interior design of the FC is pleasing.
SC7	The FC is attractively decorated.
Servicescape-Equipment / Facility Condition	
**SC8**	**The equipment used is of high quality.**
SC9	The equipment used is always in good condition.
SC10	The FC is well equipped with complementary facilities (lounge, cafeteria, etc.).
SC11	The equipment and facilities are safe.
SC12	The facilities are well maintained.
Servicescape-ambient condition	
**SC13**	**The background noise level in the FC is acceptable.**
SC14	The FC is kept clean.
**SC15**	**The music used in the FC makes the training environment a more pleasant place.**
SC16	The ambience of the FC is good.
Servicescape-facility system	
SC17	The lighting levels are adequate.
SC18	The temperature and humidity are comfortable.
SC19	The air quality is acceptable.
**SC20**	**The heating**,** ventilation**,** and air conditioning system works well.**
Perceived value	
Perceived value 1	The services in this FC are worth what they cost.
Perceived value 2	Overall, the value of the services in this FC is high.
Satisfaction centre	I am satisfied with the sports services at this FC
Loyalty	
**Loyalty 1**	**I have made positive comments to a friend about this FC**
**Loyalty 2**	**If asked**,** I will recommend this FC**
Loyalty 3	If I had to unsubscribe, I would then re-subscribe to this FC.

Bold rows correspond to predictors excluded after applying the correlation filter.
